# Physical supports from liver cancer cells are essential for differentiation and remodeling of endothelial cells in a HepG2-HUVEC co-culture model

**DOI:** 10.1038/srep10801

**Published:** 2015-06-08

**Authors:** Geraldine Giap Ying Chiew, Afu Fu, Kar Perng Low, Kathy Qian Luo

**Affiliations:** 1 School of Chemical and Biomedical Engineering, Nanyang Technological University, Singapore

## Abstract

Blood vessel remodeling is crucial in tumor growth. Growth factors released by tumor cells and endothelium-extracellular matrix interactions are highlighted in tumor angiogenesis, however the physical tumor-endothelium interactions are highly neglected. Here, we report that the physical supports from hepatocellular carcinoma, HepG2 cells, are essential for the differentiation and remodeling of endothelial cells. In a HepG2-HUVEC co-culture model, endothelial cells in direct contact with HepG2 cells could differentiate and form tubular structures similar to those plated on matrigel. By employing HepG2 cell sheet as a supportive layer, endothelial cells formed protrusions and sprouts above it. In separate experiments, fixed HepG2 cells could stimulate endothelial cells differentiation while the conditioned media could not, indicating that physical interactions between tumor and endothelial cells were indispensable. To further investigate the endothelium-remodeling mechanisms, the co-culture model was treated with inhibitors targeting different angiogenic signaling pathways. Inhibitors targeting focal adhesions effectively inhibited the differentiation of endothelial cells, while the growth factor receptor inhibitor displayed little effect. In conclusion, the co-culture model has provided evidences of the essential role of cancer cells in the differentiation and remodeling of endothelial cells, and is a potential platform for the discovery of new anti-angiogenic agents for liver cancer therapy.

Angiogenesis is one of the hallmarks in cancer. Many studies have highlighted its significance in the progression of tumor growth and metastasis^1^. Therefore anti-angiogenesis has been identified as a therapeutic approach for the treatment of many cancers. Tumor cells play important roles in angiogenesis. Many have highlighted the roles of paracrine factors in tumor-induced angiogenesis^2^,^3^, with vascular endothelial growth factor (VEGF) being the key activator in angiogenesis^4^. However, therapeutic drugs targeting VEGF molecules (Avastin) released by cancer cells, or targeting receptors on the surface of endothelial cells (ECs) (sunitinib) are not highly effective as single therapeutic agents in liver cancer^5^,^6^. In contrast, molecular agents such as sorafenib, which targets multiple signaling pathways, provide inhibition to angiogenesis and tumor growth, and have shown promising therapeutic effects against liver cancer^7^,^8^. The underlying mechanism is that common signaling pathways such as PI3K/Akt/mTOR and Ras/Raf/MEK/ERK^9^ can be activated by multiple angiogenic factors including growth factors, the extracellular matrix (ECM)^10^,^11^, integrins^11^,^12^ and other guidance molecules^12^.

One angiogenic factor that has not been investigated is the physical tumor-endothelium interactions^13^,^14^. Although several model systems have been developed that include both tumor cells and ECs, the cell lines were often cultured in spatially separated spaces in the cases of transwell chambers^2^, microfluidics^15^,^16^ and hydrogels in three-dimensional cultures^3^,^17^. Even though these systems can be used to evaluate the paracrine factors released by tumor cells on ECs, the cell-cell interactions will be hard to study in these indirect co-culture models.

Here, we present a novel co-culture model which allows direct interactions between liver cancer cells and ECs, thus facilitating the study of signaling pathways governing blood vessel formation in liver cancer. The EC used is a human umbilical vein endothelial cell line expressing a fluorescence resonance energy transfer (FRET)-based sensor for caspase-3 (HUVEC-C3), which can detect apoptosis in real time^18^,^19^. The FRET-based sensor is a recombinant DNA encoding a cyan fluorescent protein (CFP), a yellow fluorescent protein (YFP), and a 16 amino acid-peptide linker containing the cleavage sequence of caspase-3: Asp-Glu-Val-Asp (DEVD)^18^. When HUVEC-C3 cells are alive, excitation of the donor molecule (CFP) leads to the transfer of emission energy to an acceptor molecule (YFP), resulting in green fluorescence emission. When HUVEC-C3 undergo apoptosis, caspase-3 is activated which in turn cleaves the fusion protein of CFP-DEVD-YFP through its linker, abolishing the FRET effect and resulting in a change of emission fluorescence from green to blue. The liver cancer cell line HepG2-DsRed expresses a red fluorescent protein (DsRed). In this study, liver cancer cells and ECs labeled with different fluorescence proteins were cultured together to investigate their interactions. This system modeled hepatocellular carcinoma (HCC) angiogenesis much more accurately, and HUVEC-C3 differentiated only in direct contact with HepG2 cells. The physical interactions between HepG2 and HUVEC-C3 are the key factors in tilting the angiogenic balance and the cellular signaling pathways were investigated to understand the molecular mechanisms of this tumor-endothelial interaction. With the expression of a caspase-3 sensor^19^ in HUVEC-C3 cells, the survival of ECs as well as the cytotoxic effects^20^ of inhibitors and anticancer drugs were investigated concurrently.

## Results

### Co-culture of HepG2-DsRed and HUVEC-C3 result in HUVEC-C3 cells differentiation and formation of tube-like structures

**Figure 1:**
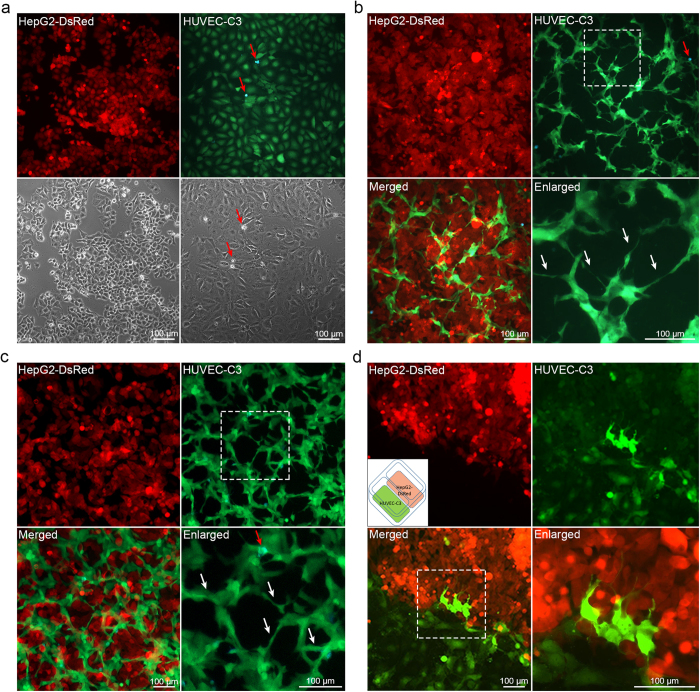
Co-culture of HepG2-DsRed and HUVEC-C3 induces HUVEC-C3 differentiation. (**a**) Epithelioid morphology of HepG2-DsRed (red) and HUVEC-C3 (green) when cultured alone. Live HUVEC-C3 cells appeared green in the FRET images, while apoptotic cells appeared blue (red arrows). (**b**) Establishing a monolayer of HepG2-DsRed followed by seeding HUVEC-C3 cells and (**c**) co-culturing both cells concurrently induced differentiation of HUVEC-C3 after two days. HUVEC-C3 showed elongation and branching (white arrows in enlarged panel), while HepG2-DsRed remained cobblestone-shaped. Few HUVEC-C3 cells undergo apoptosis (blue, red arrows) in the co-cultures. (**d**) Sprouting of HUVEC-C3 into HepG2-DsRed could be visualized when both cells were co-cultured side by side.

We utilized HUVEC-C3 cells which were stably transfected with a FRET sensor for caspase-3^19^,^21^,^22^. HUVEC-C3 cells appeared green when alive and blue (Fig. 1, red arrows) when undergo apoptosis in FRET images. Mono-cultures of HUVEC-C3 and HepG2-DsRed (red) displayed cobblestone cell morphologies, associating with each other in small islands (Fig. 11a). When HUVEC-C3 was co-cultured with HepG2-DsRed, tubular networks were observed with the differentiation of HUVEC-C3 (Fig. 1b, top right), while HepG2-DsRed remained in their cobblestone morphologies (Fig. 1b, top left). Elongation and multiple protrusions of HUVEC-C3 was observed in the co-culture (Fig. 1b, bottom right), with few cells undergoing apoptosis (Fig. 1b, top right, red arrows).

**Figure 2:**
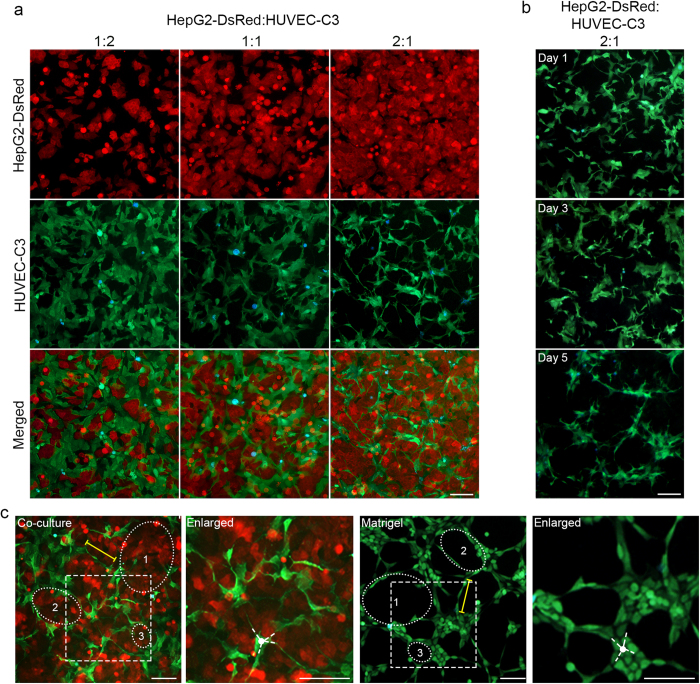
A ratio of 2:1 HepG2-DsRed: HUVEC-C3 induced high degree of differentiation in HUVEC-C3. (**a**) HepG2-DsRed (red) and HUVEC-C3 (green) were seeded at different ratios and network formations were captured at day two. Apoptotic HUVEC-C3 appeared blue under FRET imaging. 2:1 ratio of HepG2-DsRed:HUVEC-C3 co-culture showed the best tubular formation after two days, with HUVEC-C3 appearing to have the highest degree of differentiation with a high percentage of elongation of HUVEC-C3, while HUVEC-C3 were less differentiated in the other ratios of HepG2-DsRed: HUVEC-C3 co-cultures. (**b**) Tubular formation showed few network formations after one day, with cells starting to form protrusions and reorganized within the culture. Network formation was achieved after two days of co-culturing at a ratio of 2:1 of HepG2-DsRed:HUVEC-C3 (as observed in panel a). This network formation started to regress by day 5. (**c**) Comparison of the co-culture model (left) with the matrigel assay (right). Areas of tubules (circles 1, 2, and 3), average tubule length (yellow) and junctions formed (in enlarged panel) are similar in both models by a quick comparison of images.

We performed co-cultures of the cell lines in two ways: 1) establishing a monolayer of HepG2 cells, followed by the addition of HUVEC-C3 cells (Fig. 1b), and 2) co-culturing both cell lines together in a ratio of 2:1 HepG2-DsRed:HUVEC-C3 cells (Fig. 1c). No differences were observed between both methods, with equally successful differentiation and tubular networks formed (Fig. 1b, c). In order to understand the physical interactions, we looked at the contact points between both cells by seeding HepG2-DsRed and HUVEC-C3 side by side (Fig. 1d). When both cells migrated towards one another and made physical contact, HUVEC-C3 cells formed protrusions and sprouts into the HepG2-DsRed colony (Fig. 1d). Both vasculogenesis (Fig. 1b, c) and sprouting angiogenesis (Fig. 1d) which are characteristics of tumor angiogenesis^12^ were demonstrated when ECs were activated by angiogenic signals from HepG2-DsRed cells.

In addition, we performed co-cultures of other human cell lines with ECs and discovered HUVEC-C3 cells were able to differentiate with the formation of tubule-like structures in co-cultures of HUVEC-C3 cells and liver cells (Supplementary Table S1). HCC cells HepG2 and hepatic cells L0-2 were able to induce tubule formation, while other human tumor cell types such as breast adenocarcinoma MDA-MB-231 and lung carcinoma A549 were unable to induce tubule formation (Supplementary Fig. S1). Although other non-cancerous human cell types such as lung fibroblast IMR90 and embryonic kidney cells HEK-293 induced HUVEC-C3 differentiation and formed tubule networks (Supplementary Table S1), we focused on the HepG2-HUVEC-C3 co-culture as they formed aberrant and chaotic tubular networks representative of tumor angiogenesis.

Different ratios of HepG2-DsRed and ECs were tested in the co-culture models and it was found that as long as HepG2-DsRed and HUVEC-C3 cells were cultured together for a period of time (>3 days), the HUVEC-C3 cells were able to differentiate and form tubular networks (Fig. 2a and Supplementary Movie S1). However, to ensure a high degree of HUVEC-C3 differentiation and rapid formation of tubular networks, a fixed ratio of 2:1 HepG2-DsRed: HUVEC-C3 was used for the subsequent experiments.

When the co-culture cells were cultured for longer periods, HUVEC-C3 elongated and projected outwards to form tubular networks within three days, followed by regression of the networks when cultured for five days (Fig. 2b). These phenotypic characteristics were similar to the gold standard for EC differentiation, the matrigel differentiation assay, where tubule formation occurred within 5 hr (Fig. 2c) and regressed in a day.

The tubule networks in the co-culture were comparable to the networks formed when HUVEC-C3 was plated on matrigel. The average length of tubules (yellow), the junctions (indicated by the white dot in the enlarged panels), and the areas of tubules formed (circles 1, 2 and 3) were all similar to those in the matrigel (Fig. 2c). The tubule formations in the co-culture were similar to the matrigel differentiation assay, enabling it to function as a screening platform for anti-angiogenesis agents.

### Phenotypic characteristics of activated HUVEC-C3 cells induced by HepG2

**Figure 3:**
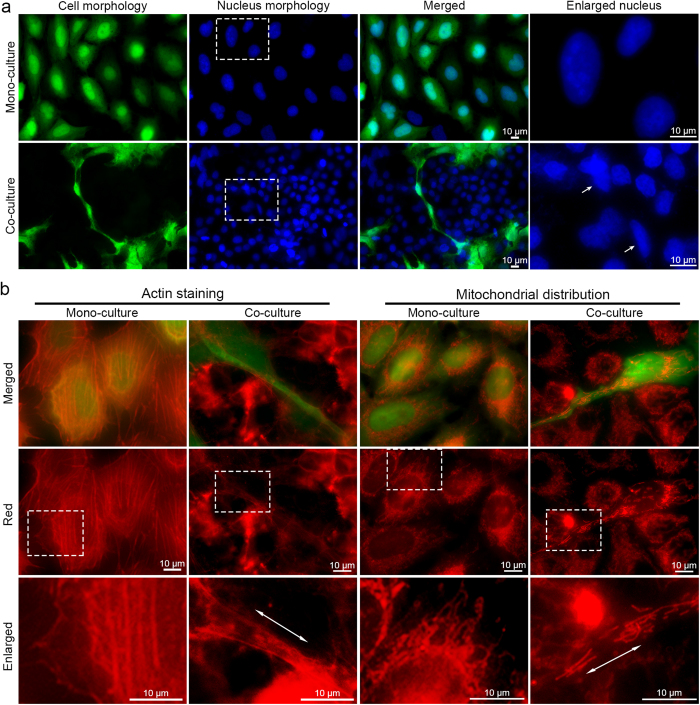
Phenotypic activation of HUVEC-C3 was revealed by immunostaining of mono- and co-cultures. Non-fluorescent HepG2 cells and HUVEC-C3 (green) were co-cultured in a ratio of 2:1 of HepG2:HUVEC-C3 for two days before fixation with paraformaldehyde and stained with the respective markers. (**a**) HUVEC-C3 (green) elongated and formed linkage with each other when co-cultured with HepG2. The nucleus (blue) was compressed from round to oval within the co-culture (white arrows, enlarged nucleus). (**b**) Phalloidin staining (red) showed reorganization of the actin filaments (left) with the actin filaments pulled along the cell (enlarged image). MitoTracker staining (red) showed redistribution of mitochondria (right) throughout the elongated HUVEC-C3 (enlarged image) upon activation by HepG2.

To understand the morphological differences in activated HUVEC-C3, we studied the phenotypic characteristics of differentiated HUVEC-C3 induced by HepG2 cells. We cultured HUVEC-C3 cells with HepG2 cells expressing no fluorescence and stained the cells with various markers. When HUVEC-C3 cells were activated by co-culturing with HepG2, HUVEC-C3 cells differentiated and elongated, forming linkages with each other (Fig. 3a). A prominent feature that was observed in differentiated HUVEC-C3 cells was the change of nuclear morphology from round to oval (Fig. 3a, enlarged nucleus), whereas in mono-culture of HUVEC-C3, the cells remained cobblestone-shaped with round nuclear morphologies. Other phenotypic changes observed on the differentiated HUVEC-C3 cells included changes to the cytoskeleton and mitochondria redistribution. Staining of phalloidin (Fig. 3b, left) and MitoTracker (Fig. 3b, right) showed the actin filaments and mitochondria of HUVEC-C3 spreading outwards along the course of its differentiated path. The phenotypic changes of HUVEC-C3 (Fig. 3a, b) were due to the interactions with HepG2 cells in the co-culture condition, as these changes were not observed in the HUVEC-C3 mono-culture, nor were they observed in the HepG2 cells in the co-culture.

### HepG2 as physical supports for HUVEC-C3 differentiation within the co-culture

**Figure 4:**
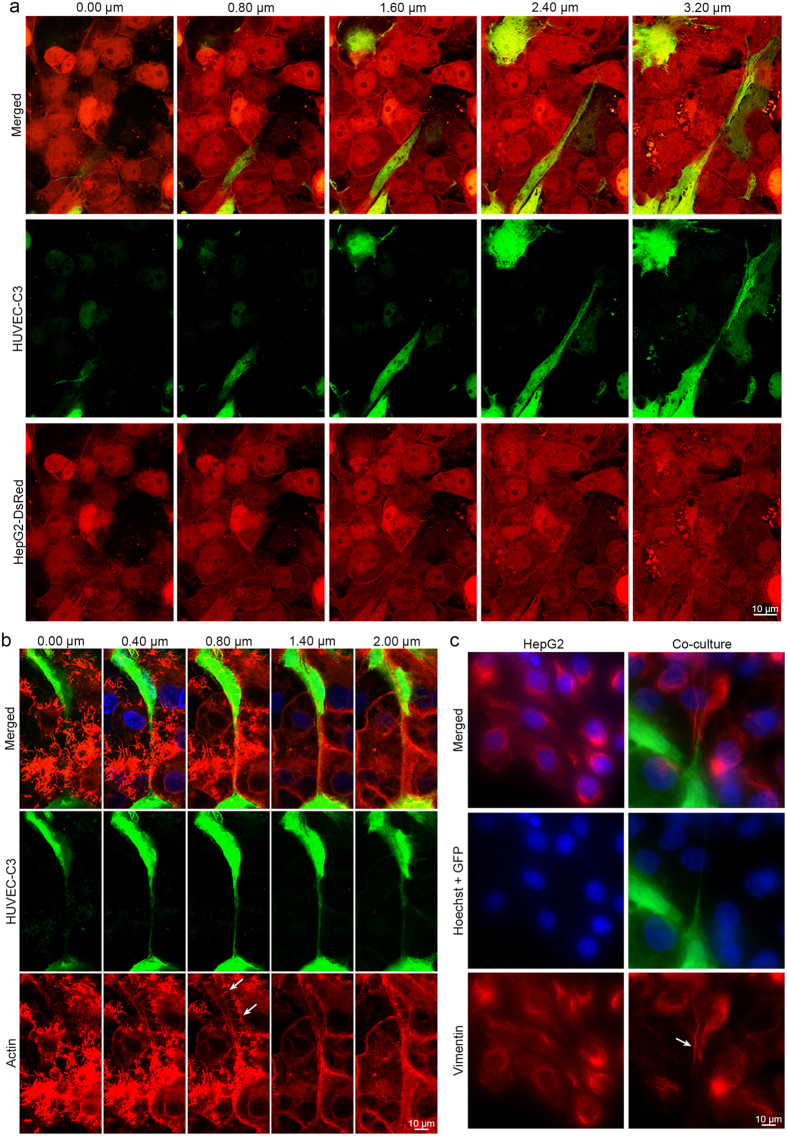
Differentiation of HUVEC-C3 (green) with the physical supports from HepG2 cells. (**a**) Co-culture of HepG2-DsRed and HUVEC-C3 for two days before fixation and visualized by confocal microscopy. Z-stack images showing the relative positions between HepG2-DsRed and HUVEC-C3. HUVEC-C3 formed protrusions and elongations above HepG2-DsRed cell sheet in co-culture. (b and c) Non-fluorescent HepG2 cells and HUVEC-C3 (green) were co-cultured in a ratio of 2:1 of HepG2:HUVEC-C3 for two days before fixation with paraformaldehyde and stained for (**b**) actin or (**c**) vimentin. Actin staining with phalloidin revealed actin networks (red) of HepG2 running perpendicularly (white arrows) under HUVEC-C3. (**c**) HepG2 cells stretched out (white arrow) to receive the elongations of HUVEC-C3 (green). Vimentin is expressed in HepG2 but not in HUVEC-C3 cells.

Next, we looked at the interactions between HepG2 and HUVEC-C3 in detail. We performed confocal imaging of the co-cultured cells and observed that HepG2-DsRed cell sheets formed the bottom layer, with part of HUVEC-C3 cells wedged between. HUVEC-C3 then elongated and formed protrusions above the layer of HepG2-DsRed cells (Fig. 4a).

Actin staining of the co-culture revealed that HepG2 cells were mainly found attached to the bottom of the plate with HUVEC-C3 cells forming networks above HepG2 cells (Fig. 4b). This phenomenon was observed regardless of how the two types of cells were seeded: in a mixture or in sequence, where HUVEC-C3 cells were seeded together with HepG2 or after a HepG2 monolayer was formed. Fig. 4b revealed that HepG2 cells supported HUVEC-C3 from underneath, where the actin filaments of HepG2 moved in perpendicular to HUVEC-C3 elongations (white arrow). Z-stack images showed the actin filaments of HepG2 cells spreading outwards, from its initial attachments on the plate, towards that of HUVEC-C3 cells and supporting it from beneath (Supplementary Movies S2 and S3).

Staining of the intermediate filament (vimentin) gave a clearer picture of how HepG2 and HUVEC-C3 interacted. Only HepG2 expressed high levels of vimentin while HUVEC-C3 cells did not, allowing us to visualize the structural changes that HepG2 cells undertook to accommodate the differentiated HUVEC-C3 cells. As shown in Fig. 4c, vimentin (red) was initially co-localized near the nucleus (blue)^23^ in a dispersed fashion in HepG2 mono-culture. This however changed in the co-culture model, where vimentin within HepG2 cells stretched towards an elongation of the HUVEC-C3 cell. Z-stack imaging (Supplementary Movies S4 and S5) showed the rims of HepG2 incurving to form a trench-liked structure, with the arms of HUVEC-C3 resting right in the middle of the curvature formed by HepG2 (Fig. 4c).

Both actin and vimentin staining revealed the cytoskeleton changes of HepG2 underwent in order to accommodate HUVEC-C3 in the formation of tubular networks. HepG2 acted as physical supports for HUVEC-C3 and there is evident interactions taking place between both cell lines, with morphological adaptations of both cells to accommodate each other.

### Physical contact is essential for HepG2-induced HUVEC-C3 differentiation

**Figure 5:**
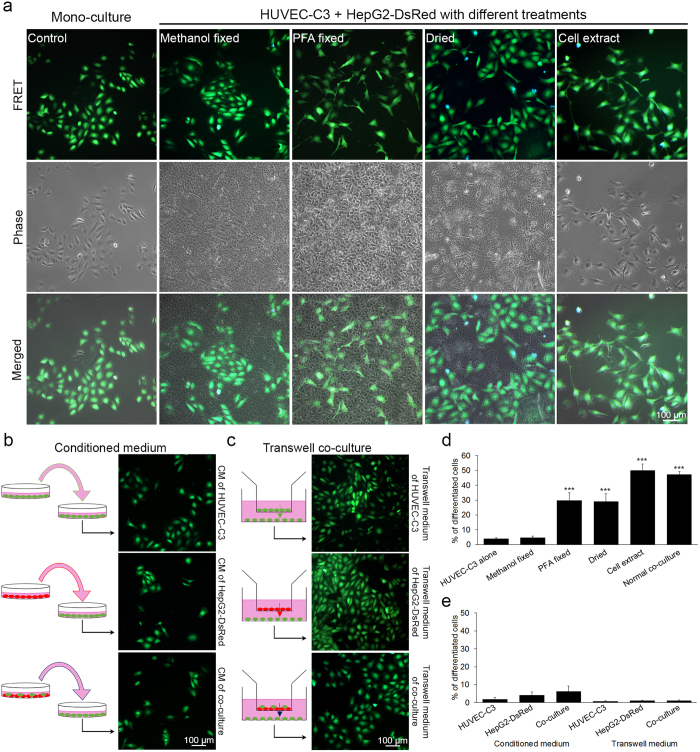
Physical contact is essential for HUVEC-C3 differentiation. (**a**) HepG2-DsRed cells were fixed with methanol, paraformaldehyde, dried rapidly, or the cell extract of HepG2-DsRed was coated on a petri dish before seeding HUVEC-C3 cells. After two days of culturing, most of the HUVEC-C3 cells remained alive (green) and did not undergo apoptosis (blue cells). (**b**) Conditioned media of HUVEC-C3, HepG2-DsRed and the co-culture were added to HUVEC-C3 cells, (**c**) or the cells were cultured in the insert of a transwell with HUVEC-C3 at the bottom of the well for two days. (**d** and **e**) The percentages of differentiated cells observed in (**a**) and (**b** and **c**) respectively were quantified from three independent experiments with at least four observation fields each. Data represents mean ± SEM. *** *p* < 0.005 *vs*. control of HUVEC-C3 alone (Student’s *t*-test).

To demonstrate that HUVEC-C3 differentiation was indeed induced by physical contacts with HepG2, we decided to inactivate the HepG2 cells while preserving their physical structures for cell-cell interactions. A monolayer of HepG2-DsRed was first established before fixing or drying the cells instantaneously, followed by culturing HUVEC-C3 cells on top of the dead HepG2-DsRed cells (Fig. 5a). The percentages of HUVEC-C3 cells that differentiated under these conditions were quantified by calculating their form factors (FF) (Fig. 5d). Differentiated cells displayed larger perimeter and smaller area, leading to a lower FF value^24^ (equivalent to 4π(area)/(perimeter)^2^). Methanol-fixed HepG2-DsRed cells did not induce any differentiation of HUVEC-C3 cells (4.6 ± 2.0%), which was similar to the control cells (3.8 ± 1.2%), however 29.7 ± 9.3% and 28.9 ± 6.6% of HUVEC-C3 cells were able to differentiate when HepG2-DsRed cells were fixed with 4% paraformaldehyde or dried respectively (Fig. 5a, d). The numbers increased to 49.8 ± 7.7% when the extract of HepG2-DsRed cells was used to coat the surface of the plate before seeding HUVEC-C3 cells. This high level of EC differentiation was also achieved when HUVEC-C3 cells were co-cultured with live HepG2-DsRed cells (47.2 ± 3.5%, Fig. 5d).

Fixation of HepG2-DsRed cells with paraformaldehyde or drying the HepG2-DsRed cells could preserve cell surface proteins necessary for inducing HUVEC-C3 differentiation. The cell extract containing the membrane proteins of HepG2-DsRed also resulted in HUVEC-C3 differentiation. However, fixation of HepG2-DsRed cells with methanol altered protein conformations and dissolved lipids which result in the removal of lipoproteins, thus preventing protein-protein interactions between HepG2-DsRed and HUVEC-C3. There is a high possibility that the protein that mediated HUVEC-C3 differentiation might be membrane proteins, including lipoproteins on the surface of HepG2 cells.

To determine whether secretory factors were necessary to induce HUVEC-C3 differentiation in the co-culture, we cultured HUVEC-C3 cells with the conditioned media of HepG2-DsRed and that of the co-culture. Furthermore, to ensure that cell densities will not affect HUVEC-C3 differentiation, we conducted the experiments with both low (2 × 10^5^ cells / 60 mm dish) and high (6 × 10^5^ cells / 60 mm dish) densities of cells. No significant differentiation was observed (Fig. 5b, e). This remained true when HUVEC-C3 cells were cultured in the bottom chamber of a transwell at densities of 0.8 × 10^4^ cells / well and 2 × 10^4^ cells / well with either HepG2-DsRed or the co-culture seeded in the transwell insert (Fig. 5c, e), allowing paracrine factors to diffuse through to the HUVEC-C3 at the bottom of the well. As no significant differentiation of HUVEC-C3 was observed in both low and high density conditions, only images of HUVEC-C3 cultured at low densities with the conditioned medium and the images of HUVEC-C3 cultured at high densities in transwell experiments are presented.

Physical contact with HepG2 cells was important for HUVEC-C3 differentiation as HUVEC-C3 cells were able to differentiate even when co-cultured with dead HepG2-DsRed cells. Release of growth factors from HepG2-DsRed cells is not the cause of HUVEC-C3 differentiation in the co-culture model.

### Investigating the angiogenic signaling pathways in the co-culture model

**Figure 6:**
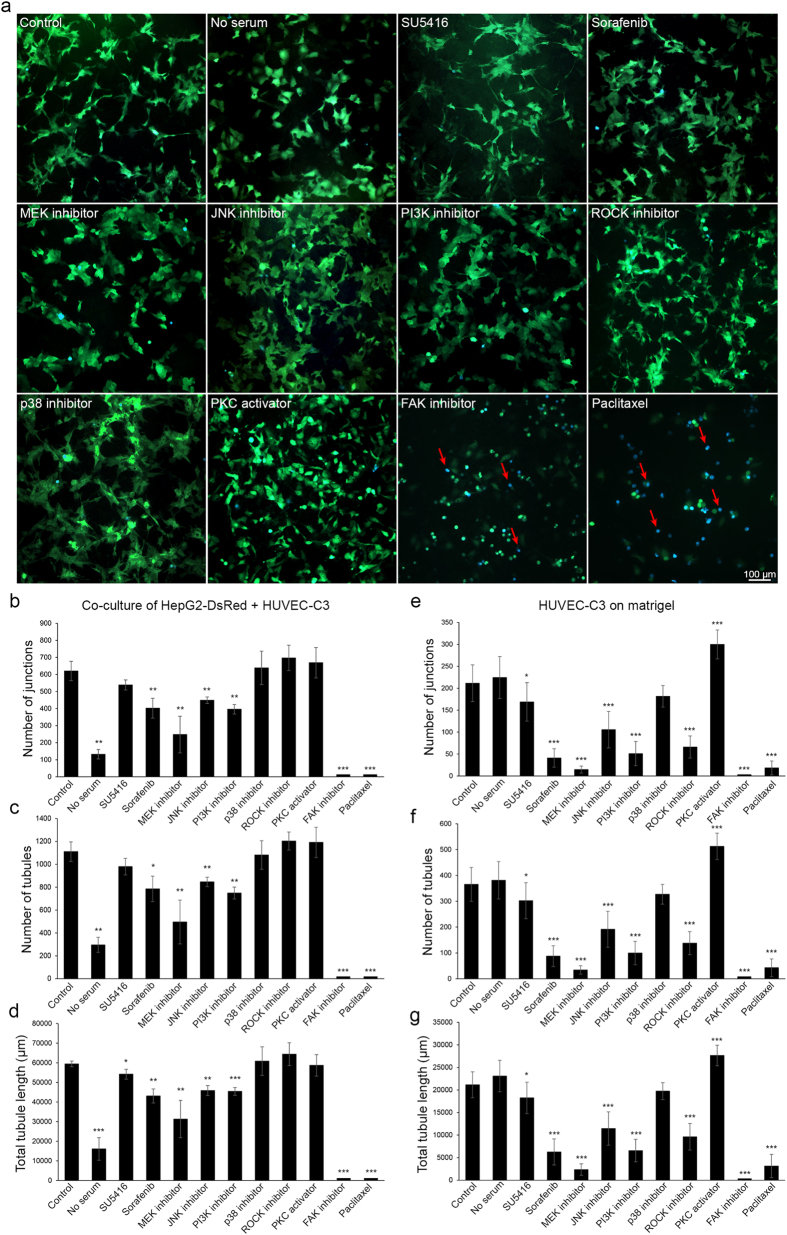
Investigation of signaling molecules important for tubule formation in the co-culture model. (**a**) 4 μM of VEGFR inhibitor (SU5416), 8 μM of sorafenib, 10 μM MEK inhibitor (U0126), 10 μM of JNK inhibitor (SP600125), 20 μM of PI3K inhibitor (LY294002), 10 μM of p38 inhibitor (SB202190), 10 μM of ROCK inhibitor (Y27632), 20 nM of PKC activator (PMA), 10 μM of FAK inhibitor (Y15) and 200 nM of paclitaxel were added to the co-culture model upon cell seeding of HepG2-DsRed (not shown) and HUVEC-C3 (green). Apoptotic HUVEC-C3 appeared blue (red arrows). (**b-g**) Quantification of tubule formation after addition of various angiogenic mediators and inhibitors in the co-culture model (left) and matrigel differentiation assay (right, refer to Supplementary Fig. S2 for images). All images were analyzed with Angiogenesis Analyzer plugin for ImageJ software. Values were normalized to the same areas of observation field. Data shown are means ± SEM of three independent experiments with 6–9 images selected at random from (**a**) and Supplementary Fig. S2 and analyzed for (**b, e**) number of junctions, (**c, f**) number of tubules, and (**d, g**) total tubule length (μm). * *p* < 0.05, ** *p* < 0.01, *** *p* < 0.005 *vs*. control.

To investigate the molecular mechanisms of HepG2-induced differentiation of HUVEC-C3, we tested the effects of no serum, inhibitors, activators and chemotherapy drugs in the co-culture model. When the cells were cultured without fetal bovine serum (FBS) for two days, HUVEC-C3 maintained their cobblestone morphology, did not differentiate nor undergo apoptosis (Fig. 6 and Supplementary Table S2). Although essential amino acids and glucose present in the medium can keep the cells alive, growth factors, proteins and a mixture of complex substances within FBS^25^ are essential for cell growth and HepG2-induced HUVEC-C3 differentiation.

We tested two anti-angiogenic agents. SU5416 is an inhibitor for vascular endothelial growth factor receptor (VEGFR) which failed clinical trial in phase 3. At 4 μM, SU5416 did not significantly reduce the tubule formation as the level of network formation averaged from three parameters (number of junctions, number of tubules and total tubule length in μm) was only reduced by 12.8% (Fig. 6 and Supplementary Table S2). At a higher concentration of 8 μM, SU5416 still did not significantly reduce the tube formation even through the compound formed crystals in the medium. In contrast, sorafenib^8^, a clinically used drug for treating HCC and is a multi-kinase inhibitor targeting Raf kinases, VEGFR and platelet derived growth factor receptor (PDGFR), reduced average network formation by 30.7% when 8 μM of the drug was used for two days (Supplementary Table S2).

MEK inhibitor (U0126) strongly reduced tubule formation by 54.2% (Supplementary Table S2). Most HUVEC-C3 did not differentiate and remained in small clusters. The MEK/ERK pathway is one of the most important signaling pathways in angiogenesis. Inhibiting the Ras/Raf/MEK/ERK pathway as demonstrated by U0126 and sorafenib (Fig. 6) prevented HUVEC-C3 cell differentiation in the co-culture model.

JNK inhibitor (SP600125) and PI3K inhibitor (LY294002) had moderate inhibitory effect on HUVEC-C3 differentiation with 24.8% and 30.8% reduction on the network formation respectively (Fig. 6 and Supplementary Table S2). Both inhibitors can regulate survival and migration^26^,^27^ of ECs which are important for angiogenesis, but are not essential for differentiation.

Three inhibitors used in this study did not affect EC differentiation in the co-culture model. p38 inhibitor (SB202190) did not reduce network formation but rather slightly increased it by 9.6% compared to the control cells at day two (Fig. 6 and Supplementary Table S2). This result is in agreement with previous findings using p38 mitogen-activated protein kinase (MAPK) inhibitors including SB202190 which showed that p38 MAPK negatively regulated EC angiogenesis^28^,^29^.

Rho-associated protein kinase (ROCK) inhibitor (Y27632) did not enhance nor suppress tubule formation compared to the control group (Fig. 6 and Supplementary Table S2). Some have shown that ROCK signaling is essential for VEGF-mediated angiogenesis^30^, while others have shown that ROCK inhibition enhanced sprouting angiogenesis^31^,^32^. However, in the co-culture model, ROCK inhibition did not altered tubule formation, as VEGF was not the key inducer in the co-culture model.

Phorbol myristate acetate (PMA), a protein kinase C (PKC) activator and tumor promoter^33^, induced morphological changes where cell elongation was observed in both HUVEC-C3 and HepG2-DsRed cells (data not shown). The tubular networks formed by HUVEC-C3 were incomplete and unconnected (Fig. 6). Although the computer software (Angiogenesis Analyzer) used in this study characterized the elongated cells as tubular networks with 4.5% increase in tubule formation (Supplementary Table S2), a closer imaging analysis revealed that they are quite different from the differentiated ECs in the control group. This may be due to the ability of PMA to cause HUVEC-C3 differentiation, and not because of their physical interactions with HepG2-DsRed cells.

Most of the HUVEC-C3 cells did not undergo apoptosis when they were treated with the aforementioned inhibitors. Less than 5% of the treated cells appeared as blue color in the FRET images (Fig. 6 and Supplementary Table S2) which indicated caspase-3 was not activated. Only focal adhesion kinase (FAK) inhibitor (Y15) and paclitaxel induced significant apoptosis in HUVEC-C3 cells (blue cells, Fig. 6) with 22.2 ± 10.3% and 24.0 ± 14.9%. The cells appeared round and no tubule formation was observed. HUVEC-C3 emitted blue fluorescence in FRET images, indicating that the FRET effect was completely abolished due to the cleavage of C3 sensor by caspase-3^18^,^19^. FAK inhibitor prevented HUVEC-C3 cells from attaching to the plate, resulting in anoikis^34^, where cells undergo apoptosis due to detachment. Paclitaxel on the other hand, triggered cell apoptosis of both HUVEC-C3 (Fig. 6) and HepG2-DsRed (data not shown), demonstrating its ability as a chemotherapeutic drug that targets all proliferating cells.

### HepG2-HUVEC-C3 co-culture as a new drug discovery model in comparison with the matrigel-based angiogenesis assay

We have shown that the co-culture model and the matrigel differentiation assay were similar in their phenotypic characteristics (Fig. 2c). Matrigel differentiation assay is the gold standard for *in vitro* angiogenesis assay, where support from an ECM is present for EC differentiation. To examine the physical support provided by HepG2-DsRed cells in the co-culture and to compare the differences between matrigel and the co-culture as functional angiogenesis assays, we applied the conditions used in the co-culture to the matrigel differentiation assay which consist of only HUVEC-C3 cells. The results showed discrepancy from the co-culture model when VEGFR, ROCK inhibitors and no serum conditions were used. HUVEC-C3 cells formed tubules under no serum conditions on the matrigel (6.7% increase in network formation, Supplementary Fig. S2 and Table S3), however differentiation of HUVEC-C3 was strongly inhibited under no serum conditions in the co-culture model (a reduction of 75.0% tubular networks, Fig. 6 and Supplementary Table S2). Matrigel, which is a tumor derived matrix, contains growth factors^10^ which were necessary for the differentiation of HUVEC-C3, thus allowing HUVEC-C3 to differentiate even without any addition of serum (Supplementary Fig. S2).

SU5416 inhibited tubule formation in the matrigel differentiation assay (a reduction of 17% tubule formed, *p* < 0.05 in all three parameters of tubule networks quantified, Fig. 6e-g), however in the co-culture model, differentiation of HUVEC-C3 was not significantly inhibited (a reduction of 12.8% tubule formed, *p *< 0.05 in only one parameter of tubule network quantified, Fig. 6d). This further confirmed that VEGF is not the key inducer of HUVEC-C3 differentiation in the co-culture model, whereas addition of SU5416 to the matrigel which contain high levels of VEGF could significantly inhibit tubule formation^35^.

ROCK inhibitor (Y27632) prevented tubule formations on the matrigel assay (a reduction of 61.8% network formation, Supplementary Table S3). This could be due to the inhibitor preventing VEGF mediated angiogenesis^30^ of ECs on the matrigel, however sustained inhibition to the ROCK signaling pathway was unable to inhibit HUVEC-C3 tubular networks formation on the co-culture as the underlying support from HepG2-DsRed cells was the key inducer in HUVEC-C3 differentiation and not that of VEGF.

To further demonstrate the importance of physical supports provided by HepG2, we applied the conditioned medium from HepG2 to the matrigel consisting of only HUVEC-C3. No significant differences were observed in the network formations when only HUVEC-C3 was used or when the conditioned medium from HepG2 was supplied to the matrigel as seen in Supplementary Fig. S3. This result indicated that the paracrine factors from the HepG2 conditioned medium were not the main angiogenic stimulants in this matrigel system.

All other inhibitors had the same suppressing effect on tubule formations both on the matrigel and the co-culture model, with inhibitors (eg, MEK, JNK and PI3K inhibitors) exhibiting their effects much more effectively on the matrigel (Fig. 6). The physical niche provided by HepG2 cells in the co-culture model protected HUVEC-C3 cells from the inhibitors treatments, which illustrated the true clinical situation in HCC patients, where anti-VEGF therapies often have transient effects with relapse and tumor progression^6^.

These results suggested that the HepG2-HUVEC co-culture model is more representative of liver cancer-induced angiogenesis. Another advantage of this model is having a FRET based-C3 sensor in the ECs, which enabled the identification of vascular disrupting agents (VDAs)^19^ and angiogenesis inhibiting agents (AIAs). Furthermore, two days of cell culturing in the co-culture model allowed the identification of VDAs such as paclitaxel, which induced HUVEC-C3 apoptosis on the co-culture (Fig. 6), and not in the matrigel assay (Supplementary Fig. S2). Survival and cytotoxicity to ECs can be monitored with the co-culture model.

### Signaling pathways undertaken by the HepG2-HUVEC-C3 co-culture model in mediating tumour angiogenesis

**Figure 7:**
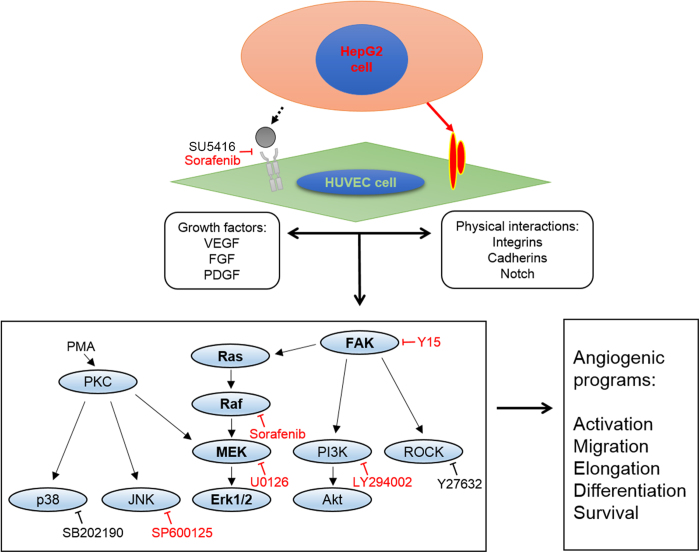
Signaling pathways and molecular mechanisms mediating HUVEC-C3 differentiation with HepG2-DsRed. Both growth factors and physical interactions are required for proper differentiation of HUVEC-C3 when co-cultured with HepG2, with various signalling pathways triggered by the receptors (left box) and the eventual result of angiogenic programming of ECs (right box). Inhibitors highlighted in red were able to suppress endothelial differentiation and tubule formation in the co-culture model. Of particular importance are the FAK and Raf/Ras/MEK/ERK pathways.

A clearer understanding of the molecular signaling pathway (Fig. 7) within the co-culture is presented with the use of inhibitors and drugs. The inhibitors of the FAK and Ras/Raf/MEK/ERK signaling pathways have prevented HUVEC-C3 differentiation and tubule formation in the co-culture model. The FAK pathway (Fig. 7) is essential for the survival of HUVEC-C3 cells and regulates many downstream signaling pathways that lead to angiogenic reprogramming within HUVEC-C3 cells. Inhibition of both the PI3K/Akt/mTOR and Ras/Raf/MEK/ERK signaling pathways have recently displayed promising treatment response to HCC^7^ which has also prevent HUVEC-C3 differentiation in the co-culture model, suggesting the potential of the co-culture model as a screening platform for HCC. On the other hand, inhibitors that could only prevent HUVEC-C3 differentiation in the co-culture and not the matrigel system might not be highly relevant in tumor angiogenesis, where the VEGFR^36^ and ROCK^30^ inhibitors have failed to elicit an inhibitory response in the co-culture.

## Discussion

EC morphogenesis in liver engineering have been demonstrated by many^13^,^37^,^38^. However, most focused on liver tissue engineering^37^,^38^ or introducing new protocols^13^ to stimulate tumor angiogenesis. Here, we showed that in a co-culture of liver cancer cells and ECs, the ECs were able to undergo morphogenesis, without the use of ECM components or 3D models. This is similar to the layered co-culture of hepatocytes and ECs employed by Harimoto *et al*. and Takayama *et al*. where both studies focused on the up-regulation of liver functions^39^ and liver specific genes^40^. However, both did not realized EC morphogenesis or tubular network formations may be induced by the liver cell sheets. Few had studied the heterotypic interactions between liver cells and ECs where liver cells may possess an innate ability to induce EC angiogenesis.

We are the first to report the hepatocyte-EC co-culture model as a two-dimensional platform for studying liver cancer-induced angiogenesis. Interestingly, the aberrant tubular networks formed in this co-culture model is comparable to those *in vivo*, where intravital optical imaging of neovascularization of the liver tumor^41^ and other animal models^42^ showed similar chaotic and abnormal network formation. We demonstrated the inhibition of the networks with various inhibitors, and as such dissected the molecular signaling pathways in HUVEC-C3 differentiation and presented the co-culture as a drug screening platform for HCC. VDAs and AIAs can be identified, while the survival and cytotoxicity to ECs can be investigated simultaneously.

The role of liver cancer cells as physical supports in the activation of ECs is also suggested here. In contrast to studies performed by Khodarev *et al*., paracrine factors were the key inducer in HUVEC differentiation where indirect co-culture resulted in phenotypic activation of HUVECs cultured with human glioma cells^2^. Instead of focusing on paracrine factors, we studied the cell-cell interactions in this novel co-culture model. The physical contacts and not the paracrine factors were the key to EC differentiation. Although the exact mechanism in which HepG2 and HUVEC-C3 cells interact is still unknown, the purpose of this study is to provide evidence that liver cancer cells contributed as physical supports in liver-induced angiogenesis. Characterizing the phenotypic changes in both HUVEC-C3 and HepG2 cells led to valuable insights demonstrating the crosstalk between HepG2 and ECs. The next steps would be to study the upregulated cell surface proteins in HUVECs as detected by Takayama *et al*.^40^, where integrin may mediate the interactions between hepatocytes and ECs^40^. We have also shown that FAK inhibitor targeting the membrane protein integrin was able to disrupt the differentiation of HUVEC-C3 and tubule formation in the co-culture. The cell surface markers possessed by HUVEC-C3 and the reciprocal partners on HepG2 which mediated EC morphogenesis and tubular formation are the ultimate targets to understand liver cancer-induced angiogenesis. Recognizing the critical role of liver cancer cells as physical supports to EC differentiation would bring new perspectives in liver cancer-induced angiogenesis.

Although it is not known whether this mechanism is distinct to only HCC angiogenesis or not, the significance of tumor cells as physical supports to tumor angiogenesis should not be overlooked. Importantly, many cell lines which we have tested such as the human embryonic kidney HEK293 and human neuroblastoma SK-N-SH (Supplementary Table S1), have the ability to induce EC morphogenesis, showing that this EC differentiation observed in the co-culture model may not be unique to only liver cells. The differences between the normal and tumor tubular networks induced by the participating cells should be taken into account (Supplementary Fig. S1). It should also be noted that the kidney, brain and liver cells which were able to induce EC morphogenesis are those highly vascularized *in vivo*^43^. An invaluable technique is presented here which allows further understanding of angiogenesis from a new context.

The co-culture model provides an insight for the examination of physical cues from tumor cells. To investigate tumor-associated blood vessels, the participation of cancer cells is necessary. Matrigel does not provide a platform for the association of cancer cells and may not be representative of the tumor microenvironment. Furthermore, differentiation induced by the matrigel is not specific, where non-ECs such as HepG2 and MDA-MB-231 cells^44^ are also able to form tubule-like networks.

In summary, the co-culture model is able to reflect the microenvironment where ECs and liver cancer cells co-exist, resulting in the reprogramming of various signal transduction pathways, and inducing tubule formations resembling that of the tumor vasculatures. Furthermore, to successfully inhibit tumor growth, chemotherapeutic drugs that target multiple cell types simultaneously are necessary. The co-culture model provides an excellent platform for monitoring real-time apoptosis and inhibition of neovascularization in HCC. New therapeutic strategies for HCC can be identified by targeting both tumor and ECs simultaneously.

## Material and Methods

### Reagents

The human hepatocellular carcinoma cell line, HepG2, was a generous gift from Assistant Prof. Sierin Lim, and human umbilical vein endothelial cell, HUVEC, was purchased from ATCC (Manassas, VA, USA). Matrigel was purchased from BD Biosciences (San Jose, CA, USA). All drugs and inhibitors were purchased from Calbiochem (San Diego, CA, USA). Culture media and all antibiotics were purchased from Invitrogen (Carlsbad, CA, USA).

### Cell culture

HUVEC-C3 was generated by transfecting the plasmid DNA of a FRET-based sensor C3 into HUVEC cells and isolated from a single clone^19^. HepG2-DsRed was generated by transfecting the plasmid DNA of pDsRed1-N1 vector (Clontech, Heidelberg, Germany) into HepG2 cells and isolated from a single clone stably expressing the DsRed1 protein. All mono- and co-cultures were maintained in Dulbecco’s modified Eagle medium (DMEM, GIBCO) supplemented with 10% fetal bovine serum (FBS, Hyclone, Logan, USA) and 1% penicillin/streptomycin at 37 °C and 5% CO_2._

### HepG2-DsRed and HUVEC-C3 co-culture assay

HepG2-DsRed and HUVEC-C3 cells were harvested by trypsin treatment and mixed in ratios of 1:1, 1:2 and 2:1 of HepG2-DsRed:HUVEC-C3 before seeding them in 12-well plates (3 × 10^5^ cells/well). Or, a HepG2-DsRed monolayer consisting of 2 × 10^5^ cells was established before seeding 1 × 10^5^ HUVEC-C3 cells in the 12-well plate. The medium was replaced every day in both methods. Alternatively, for side-by-side co-culture experiments, where HepG2-DsRed and HUVEC-C3 were located adjacent to each other, the cells were seeded into two separate compartments of an Ibidi culture insert (Ibidi, Martinsried, Germany) to generate a 500 ± 50 μm gap in between. When cells reached confluence, the culture insert was removed and cells were allowed to grow towards each other for two days.

### Immunofluorescence staining

Non-fluorescent HepG2 cells were used for all immunofluorescence staining of co-culture with HUVEC-C3. Vimentin (Abcam, Cambridge, UK) staining was performed to co-cultures of HepG2 and HUVEC-C3 at a ratio of 2:1. Both cell lines were seeded together and cultured for two days before fixation with 4% paraformaldehyde for 10 min at room temperature. Cells were permeabilized with 0.2% Triton-X 100 for 15 min followed by blocking with 3% BSA/0.3 M glycine/0.1% Triton-X 100 in PBS for 1 hr. After blocking, the cells were incubated at 4 ^o^C overnight with the primary antibody (1:100 for vimentin), followed by rhodamine-conjugated secondary antibody (1:100) for 1 hr at room temperature. The nucleus was stained with Hoechst 33342 before cells were mounted onto a coverslip using Mowiol^®^ 4–88 (Calbiochem).

Cells were stained for actin after paraformaldehyde fixation using phalloidin-rhodamine (Invitrogen) dissolved in methanol for 30 min. Mitochondria staining was performed using MitoTracker^®^ Red CMXRos (Invitrogen) at 100 nM for 15 min, followed by fixation with paraformaldehyde. Images were captured with a LSM 710 META laser scanning confocal microscope (Carl Zeiss, Thornwood, NY, USA).

### HUVEC-C3 differentiation with various treatments to HepG2-DsRed monolayer

HepG2-DsRed cells were seeded in a 10 cm petri dish and allowed to grow to confluence before fixation or dried. 100% cold methanol was used to fix the monolayer for 10 min on ice, or 4% paraformaldehyde solution was added for 15 min at room temperature. The monolayer was then washed thrice with PBS for 10 min each. Alternatively, the monolayer was rinsed rapidly with autoclaved Millipore water and removed of all solutions before drying at 37 ^o^C. 5 × 10^5^ HUVEC-C3 cells were then seeded on the monolayer. For HepG2-DsRed cell extract, HepG2-DsRed was scraped off from 10 cm petri dish and washed with PBS, followed by the addition of autoclaved Millipore water and sonication before centrifuging at 13,000 rpm for 10 min. The supernatant was then passed through a 0.2 μm filter and coated on a new 60 mm petri dish for 30 min, which was subsequently allowed to dry for another 30 min before seeding 2 × 10^5^ HUVEC-C3 cells.

### Transwell co-culture

1.5 × 10^4^ cells of HUVEC-C3 (control), HepG2-DsRed, or a mixture of HepG2-DsRed and HUVEC-C3 at the ratio of 2:1 were seeded in each transwell insert with a pore size of 0.4 μm in 24-well plates (Costar, Cambridge, UK), with 0.8 × 10^4^ (low density) or 2 × 10^4^ (high density) HUVEC-C3 cells seeded at the bottom chamber. The medium in the insert was changed every day for five days, while the medium in the bottom chamber was changed once every two days.

### Conditioned medium co-culture

Conditioned medium was collected from HUVEC-C3 or HepG2-DsRed or HepG2-DsRed-HUVEC-C3 co-culture and centrifuged at 3000 rpm for 3 min. The conditioned medium was then transferred to 2 × 10^5^ (low density) or 6 × 10^5^ (high density) HUVEC-C3 cells seeded in 60 mm petri dishes each day for five days consecutively.

### Cell shape analysis for HUVEC-C3 differentiation

Differentiation of cells were quantified with a modification to the form factor (FF) published by Mendez *et al*.^24^. Briefly, cell shapes were measured to obtain the FF value equivalent to 4π(area)/(perimeter)^2^. Differentiated HUVEC-C3 cells displayed larger perimeter and smaller area resulting in smaller FF values compared to control cells. A normal distribution of 120 control cells was plotted to find the margin in which 5% of the cells lie. This margin was taken as the basal value where HUVEC-C3 cells with an FF value smaller than the basal value was considered as differentiated cells.

### Inhibition of HUVEC-C3 differentiation with target agents

VEGFR inhibitor (SU5416), anti-HCC drug (sorafenib), MEK inhibitor (U0126), JNK inhibitor (SP600125), PI3K inhibitor (LY294002), p38 inhibitor (SB202190), ROCK inhibitor (Y27632), PKC activator (PMA), FAK inhibitor (Y15) were used to target various signaling pathways regulating angiogenesis. All inhibitors were tested for a series of concentrations. The optimal concentrations were used for each inhibitor to compare their effects on HUVEC-C3 cell differentiation, with LY294002 at 20 μM, PMA at 20 nM, SU5416 at 4 μM, sorafenib at 8 μM, while the rest were used at a concentration of 10 μM. A chemotherapy drug paclitaxel was used at a concentration of 200 nM. Randomly selected fields of view were photographed two days post seeding of the co-cultures and images were quantified by the Angiogenesis Analyzer plugin for ImageJ.

### Matrigel differentiation assay

The conditions used in the co-culture assay was also applied to the matrigel differentiation assay. Matrigel (BD Biosciences) was mixed 1:1 with pre-cooled fresh medium and coated on 24 well plates. HUVEC-C3 cells pre-treated with various inhibitors or drugs for 3 hr were seeded into each wells (8 × 10^4^ cells per well) and incubated for 5 hr along with the inhibitors or drugs. For no serum and conditioned medium conditions, matrigel was mixed 1:1 with DMEM containing no serum or the conditioned medium from HepG2-DsRed and coated on the dish, before culturing HUVEC-C3 with the respective medium. Tubular networks were then quantified with Image J (NIH, Bethesda, MD, USA), Angiogenesis Analyzer plugin.

### Image analysis

All images were captured with an AxioCam MRm camera attached to an Axio Observer.Z1 microscope (Carl Zeiss) unless otherwise stated. Images were adjusted with Image Pro Plus 6.0 using an Angiogenesis Macro downloadable from the Media Cybernetics website to obtain skeletonized images (Supplementary Fig. S4, top right), which was then analyzed by the Angiogenesis Analyzer plugin with ImageJ software (bottom left) written by Carpentier G. (2012),^45^ downloadable from the National Institute of Health website. From the analysis, number of junctions was taken for calculation, number of branches plus number of segments were equivalent to number of tubules, while total branching lengths were equivalent to tubules length (Supplementary Fig. S4)

### Statistical analysis

All data are presented as the mean ± SEM from three independent experiments. The significance between two groups were assessed by the Student’s *t*-test (two-tailed) in all experiments using Microsoft Excel. *p < 0.05 was considered statistically significant.

## Additional Information

**How to cite this article**: Chiew, G. G. Y. *et al*. Physical supports from liver cancer cells are essential for differentiation and remodeling of endothelial cells in a HepG2-HUVEC co-culture model. *Sci. Rep*. **5**, 10801; doi: 10.1038/srep10801 (2015).

## Supplementary Material

Supplementary Information

Supplementary Movie S1

Supplementary Movie S2

Supplementary Movie S3

Supplementary Movie S4

Supplementary Movie S5
