# Supplementary material for: Physical supports from liver cancer cells are essential for differentiation and remodeling of endothelial cells in a HepG2-HUVEC co-culture model
Source: Sci Rep. 2015 Jun 8;5:10801. doi: 10.1038/srep10801 (PMC4459107; doi:10.1038/srep10801)
Supplement: Supplementary Information [file srep10801-s1.pdf]

Supplementary Information

Physical supports from liver cancer cells are essential for differentiation and remodeling of endothelial cells in a HepG2-HUVEC co-culture model

Geraldine Giap Ying Chiew[+], Afu Fu[+], Kar Perng Low and Kathy Qian Luo*

School of Chemical and Biomedical Engineering, Nanyang Technological University, Singapore

[+] These authors contributed equally to this work.

* Correspondence and requests for materials should be addressed to K.Q.L.

(kluo@ntu.edu.sg).

Supplementary Figure S1

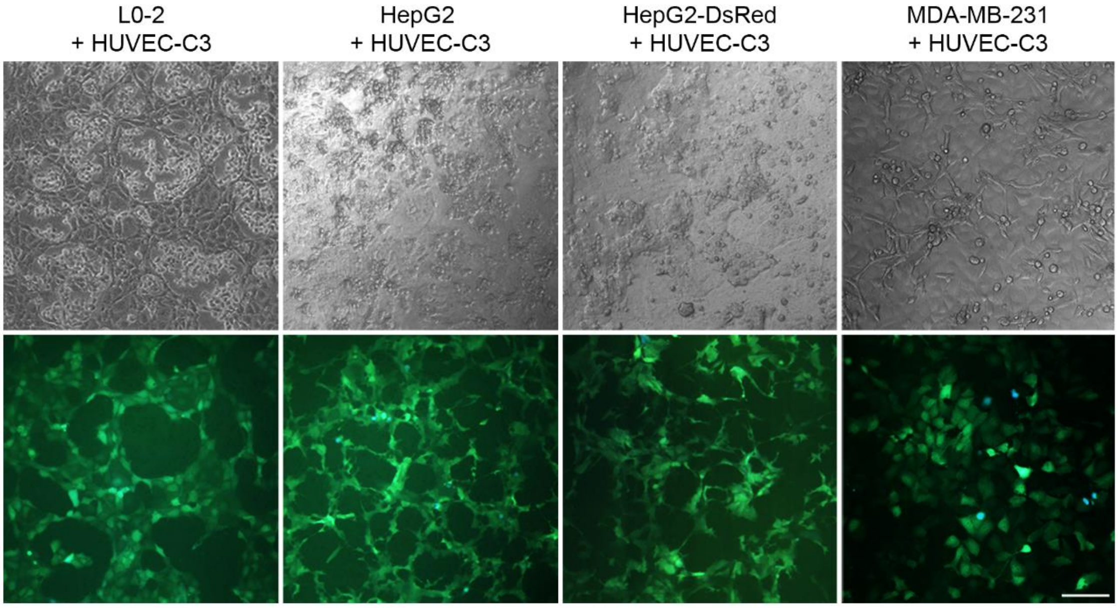

Figure S1 | Co-culture of liver cancer cells and HUVEC-C3 induces HUVEC-C3 differentiation. Only liver cell types (L0-2 and HepG2) were able to induce tubular networks, while other cell types such as breast cancer cell MDA-MB-231 were unable to form network formations. HUVEC-C3 co-cultured with L0-2 hepatic cells were able to form orderly networks compared to HepG2.

Supplementary Table S1 | Evaluation of EC differentiation and tubule formation in co-culture of HUVEC-C3 and other cells

Cell line	Cell type	Tissue	Disease	HUVEC-C3 differentiation	Tubule formation
HepG2	Human epithelial	Liver	Hepatocellular carcinoma	Yes	Yes
HepG2-DsRed	Human epithelial	Liver	Hepatocellular carcinoma	Yes	Yes
SK-N-SH	Human epithelial	Brain	Neuroblastoma	Yes	No
A549	Human epithelial	Lung	Carcinoma	No	No
MDA-MB-231	Human epithelial	Breast	Adenocarcinoma	No	No
BT-474	Human epithelial	Breast	Ductal carcinoma	No	No
L0-2	Human epithelial	Liver	Normal	Yes	Yes
HEK-293	Human epithelial	Kidney	Normal	Yes	Yes
IMR90	Human fibroblast	Lung	Normal	Yes	Yes
VSMC	Human smooth muscle	Vascular smooth muscle	Normal	Yes	No

Supplementary Table S2 | Quantification of tubule network formation under different conditions in the co-culture system

	Number of junctions	Number of tubules	Total tubule length (µm)	Average network changes	Apoptotic cells (%)	AIA/ VDA
Control	620 ± 56 (100%)	1,111 ± 85 (100%)	59,426 ± 1459 (100%)	0.0%	< 5	-
No serum	132 ± 28 (21.3%)	296 ± 66 (26.6%)	16,085 ± 5,759 (27.1%)	-75.0%	< 5	AIA
VEGFR inhibitor SU5416 4 µM	539 ± 29 (86.9%)	980 ± 72 (88.2%)	54,156 ± 2,519 (86.6%)	-12.8%	< 5	AIA
Sorafenib 8 µM	402 ± 58 (64.8%)	785 ± 112 (70.7%)	43,074 ± 3,588 (72.5%)	-30.7%	< 5	AIA
MEK inhibitor (U0126) 10 µM	248 ± 107 (40.0%)	496 ± 192 (44.6%)	31,318 ± 9,503 (52.7%)	-54.2%	< 5	AIA
JNK inhibitor (SP600125) 10 µM	449 ± 19 (72.4%)	846 ± 41 (76.1%)	45,861 ± 2,547 (77.2%)	-24.8%	< 5	AIA
PI3K inhibitor (LY294002) 20 µM	396 ± 28 (63.9%)	749 ± 52 (67.4%)	45,384 ± 1,998 (76.4%)	-30.8%	< 5	AIA
p38 inhibitor (SB202190) 10 µM	697 ± 75 (112.4%)	1,203 ± 80 (108.2%)	64,387 ± 5,801 (108.3%)	+9.6%	< 5	AIA
ROCK inhibitor (Y27632) 10 µM	638 ± 98 (101.2%)	1,081 ± 125 (97.3%)	60,835 ± 7,288 (102.4%)	+0.3%	< 5	AIA
PKC activator (PMA) 20 nM	668 ± 89 (107.7%)	1,191 ± 133 (107.2%)	58,677 ± 5,484 (98.7%)	+4.5%	< 5	AIA
FAK inhibitor (Y15) 10 µM	0	0	0	-100.0%	22.2 ± 10.3	VDA
Paclitaxel 200 nM	0	0	0	-100.0%	24.0 ± 14.9	VDA

Supplementary Figure S2

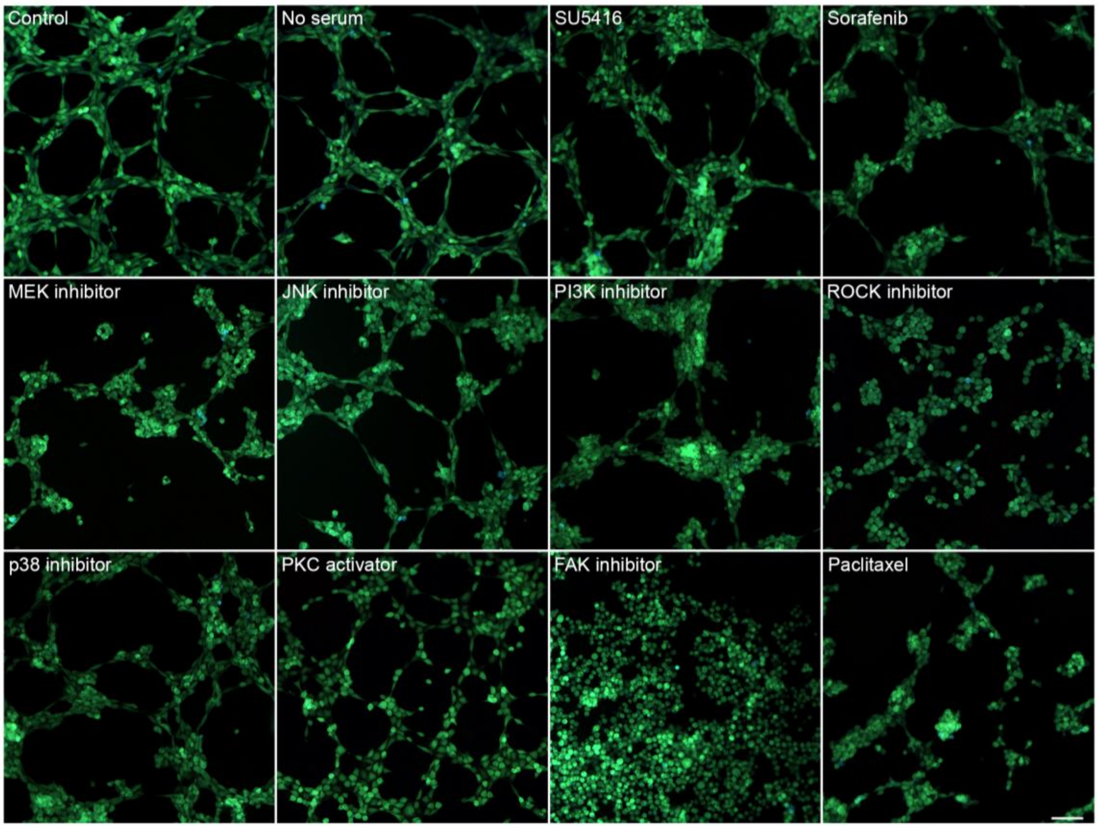

Figure S2 | Investigation of signaling molecules important for tubule formation in the matrigel differentiation assay. AIAs and VDAs used in the co-culture model were used in the matrigel differentiation assay. HUVEC-C3 was pretreated with various agents for 3 hr and during the whole assay. 4 μM of VEGFR inhibitor (SU5416), 8 μM of sorafenib, 10 μM of MEK inhibitor (U0126), 10 μM of JNK inhibitor (SP600125), 20 μM of PI3K inhibitor (LY294002), 10 μM of p38 inhibitor (SB202190), 10 μM of ROCK inhibitor (Y27632), 200 nM of PKC activator (PMA), 10 μM of FAK inhibitor (Y15) and 200 nM of paclitaxel were added to the HUVEC-C3 seeded on matrigel. Images were taken 5 hr after seeding of HUVEC-C3 on the matrigel.

Supplementary Table S3 | Quantification of tubule network formation under different conditions in the matrigel system

	Number of junctions	Number of tubules	Total tubule length (μm)	Average network changes	Apoptotic cells (%)	AIA/ VDA
Control	211 ± 42 (100%)	365 ± 66 (100%)	21,169 ± 2,864 (100%)	0.0%	< 5	-
No serum	225 ± 48 (106.6%)	381 ± 73 (104.4%)	23,102 ± 3,492 (109.1%)	+6.7%	< 5	AIA
VEGFR inhibitor SU5416 4 μM	169 ± 44 (80.1%)	302 ± 70 (82.8%)	18,247 ± 3,502 (86.2%)	-17.0%	< 5	AIA
Sorafenib 8 μM	41 ± 21 (19.4%)	87 ± 40 (23.8%)	6,275 ± 2,889 (29.6%)	-75.7%	< 5	AIA
MEK inhibitor 10 μM (U0126)	15 ± 8 (7.1%)	34 ± 17 (9.3%)	2,373 ± 1,274 (11.2%)	-90.8%	< 5	AIA
JNK inhibitor 10 μM (SP600125)	106 ± 42 (50.2%)	191 ± 70 (52.3%)	11,472 ± 3,686 (54.2%)	-47.8%	< 5	AIA
PI3K inhibitor 20 μM (LY294002)	51 ± 28 (24.2%)	100 ± 45 (27.4%)	6,581 ± 24,97 (31.1%)	-72.4%	< 5	AIA
p38 inhibitor 10 μM (SB202190)	182 ± 25 (86.3%)	327 ± 38 (89.6%)	19,746 ± 1,883 (93.3%)	-10.3%	< 5	AIA
ROCK inhibitor 10 μM (Y27632)	66 ± 25 (31.3%)	138 ± 45 (37.8%)	9,630 ± 2,954 (45.5%)	-61.8%	< 5	AIA
PKC activator 20 nM (PMA)	300 ± 33 (142.2%)	513 ± 133 (140.5%)	27,663 ± 2,282 (130.7%)	+37.8%	< 5	AIA
FAK inhibitor 10 μM (Y15)	0	0	0	-100.0%	< 5	VDA
Paclitaxel 200 nM	18 ± 16 (8.5%)	43 ± 34 (11.8%)	3,159 ± 2,593 (14.9%)	-88.3%	< 5	VDA

Supplementary Figure S3

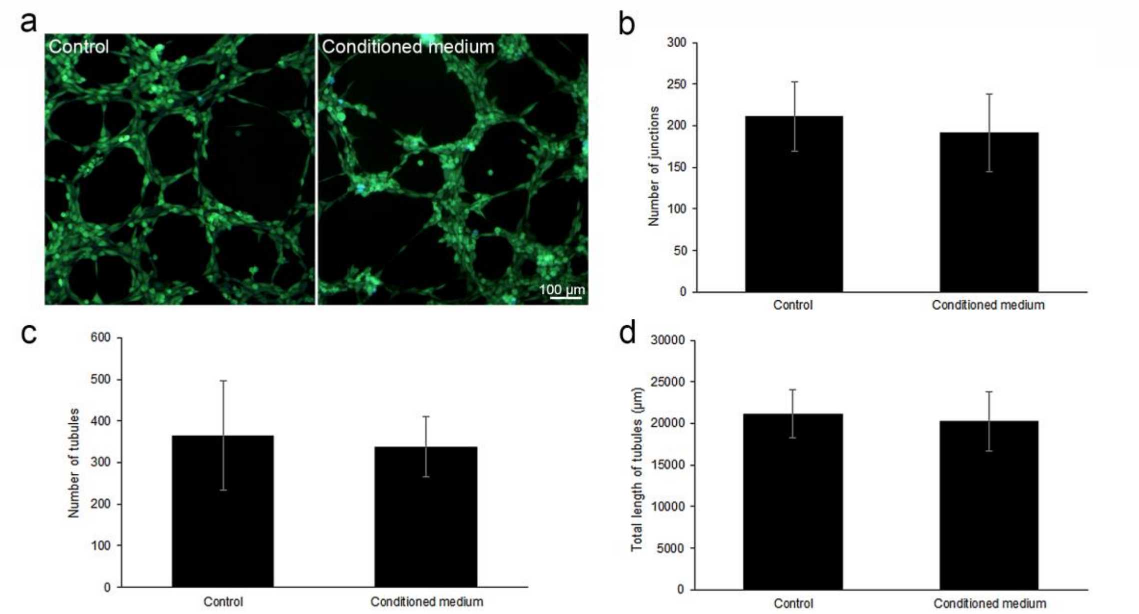

Figure S3 | Comparison of tubule formation in the matrigel differentiation assay without (control) or with the conditioned medium of HepG2-DsRed. (a) DMEM or conditioned medium from HepG2-DsRed was mixed 1:1 in matrigel and coated on petri dish, followed by seeding of HUVEC-C3 with DMEM or the conditioned medium. Images were taken 5 hr after seeding of HUVEC-C3 on the matrigel. Both control and the conditioned medium of HepG2-DsRed showed tubule formation in the matrigel assay. (b-d) Tubule formation was quantified from (a) and data shown are means ± SEM of three independent experiments with 6-9 images selected at random and analyzed for (b) number of junctions, (c) number of tubules, and (d) total length of tubules (μm).

Supplementary Figure S4

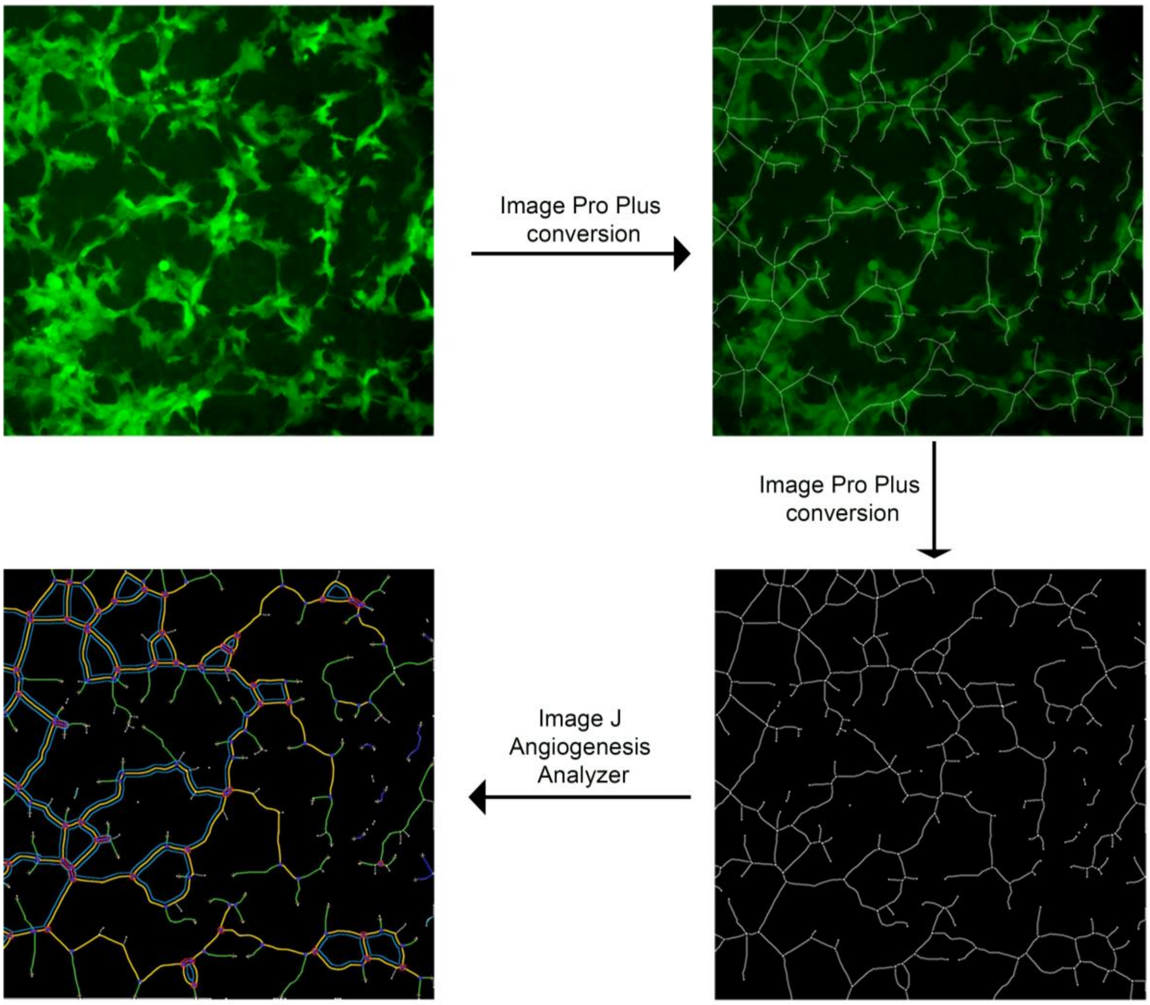

Figure S4 | Quantification of co-culture images from raw image. Images were skeletonized using Image Pro Plus angiogenesis macro followed by analyzing in Image J Angiogenesis Analyzer plugin. Green = branches, orange = master segments, red surrounded by dark blue = junctions.

Description of Supplementary Movies

Movie S1 | Time lapse of co-culture of HepG2-DsRed and HUVEC-C3. HUVEC-C3 (green) starts forming elongations and protrusions after ~7 h in culture with HepG2-DsRed cells (red). The movie was recorded in real time for 20 h after seeding the cells at maximum confluence on a petri dish at a ratio of 2:1 of HUVEC-C3 and HepG2-DsRed. HUVEC-C3 cells undergoing apoptosis (cells turning from green to blue, red arrows) can also be visualized with FRET images. The scale bar is shown in the movie.

Movie S2 and S3 | Z-stacked images of HepG2 with HUVEC-C3 co-culture for visualization of actin filaments. Non-fluorescence HepG2 was co-cultured with HUVEC-C3 (green) and stained for actin filaments (red). Actin filaments from HepG2 moved perpendicularly underneath HUVEC-C3 cells with elongations (white arrows). HepG2 cells provide support for HUVEC-C3 from below. The scale bar is shown in the movie.

Movie S4 and S5 | Z-stacked images of HepG2 with HUVEC-C3 co-culture for visualization of vimentin intermediate filament. Non-fluorescence HepG2 was co-cultured with HUVEC-C3 (green) and stained for vimentin (red). HepG2 expresses vimentin protein and not HUVEC-C3.Vimentin within HepG2 cells stretched towards an elongation of the HUVEC-C3 cell (green). The rims of HepG2 formed a trench-liked structure (white arrow), with the arms of HUVEC-C3 resting right in the middle of the curvature formed by HepG2. Other HepG2 cells not involved with HUVEC-C3 differentiation do not display this

phenomenon, with vimentin localized near the nuclei in a dispersed fashion. The scale bar is shown in the movie.
